# Real-time resolution of VP ellipsis ambiguity and processing depth

**DOI:** 10.3758/s13423-026-02873-z

**Published:** 2026-02-25

**Authors:** Hiroki Fujita, Masaya Yoshida

**Affiliations:** 1 Department of Linguistics, University of Potsdam, Potsdam, Germany; 2 Departament de Filologia Catalana, Universitat Autònoma de Barcelona, Barcelona, Spain; 3 Institució Catalana de Recerca i Estudis Avançats (ICREA), Barcelona, Spain

**Keywords:** Ellipsis, Ambiguity resolution, Sentence processing, Processing depth

## Abstract

In the VP ellipsis sentence “Bill liked himself, and John did too,” the second conjunct “John did too” can be interpreted in two ways: either as “John liked Bill too” (strict interpretation) or “John liked himself too” (sloppy interpretation). Previous research has yielded inconclusive findings regarding which interpretation is preferred during real-time sentence processing. To investigate potential causes of these mixed findings, we conducted three reading experiments (Experiments 1–3) testing whether the inconclusive results could be attributed to (i) insufficient statistical power, (ii) limitations in experimental materials, or (iii) the influence of processing depth. We also examined whether form mismatches (e.g., “herself/himself”) affect the resolution of VP ellipsis ambiguities, as in “Mary liked herself, and John did [_ellipsis_ like himself] too”. Experiment 1, with a large sample size (360 participants), showed no clear preference for either interpretation. Experiment 2, using revised materials, revealed a preference for the strict interpretation. Experiment 3 included comprehension questions targeting the interpretation of the elided material to assess the influence of processing depth. Results indicated that participants who answered correctly preferred the strict interpretation, while those who answered incorrectly preferred the sloppy interpretation. Across all three experiments, form mismatches produced no clear effect. We propose an account of the mechanisms underlying the real-time resolution of VP ellipsis ambiguities and discuss how processing depth influences preferences for the strict or sloppy interpretation.

## Introduction

This paper investigates the processing of sentences involving *ellipsis*—a phenomenon in which a sentence appears to lack surface material that is necessary for conveying its meaning. For example, consider the following sentence:

- (1) Bill liked Mary, and John did too.

**Fig. 1:**
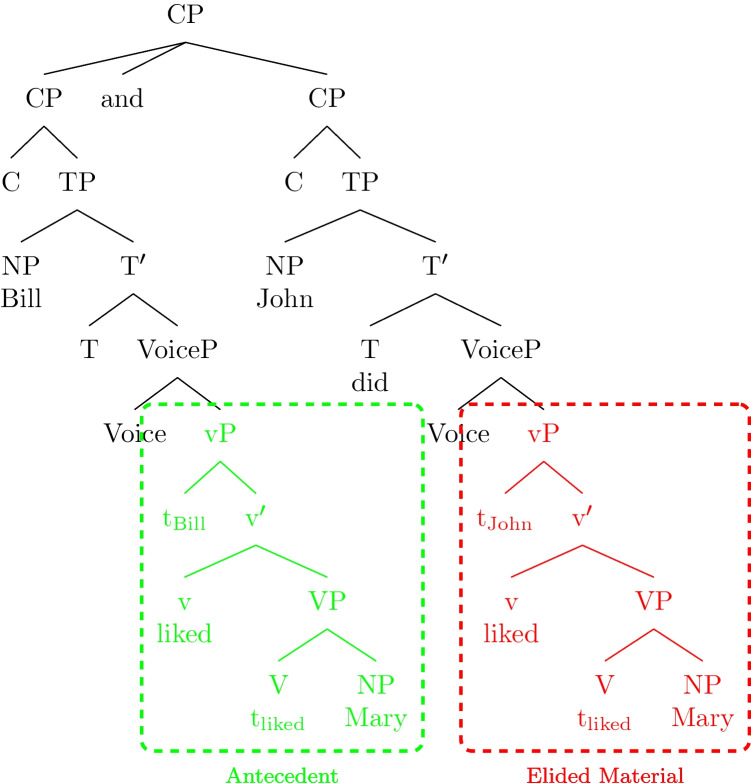
Syntactic structure of sentence (1)

In (1), “*John did too*” is interpreted as “*John liked Mary too,*” but words that are necessary to express this meaning—*liked* and *Mary*—are missing. One analysis of this phenomenon is that the ellipsis site “*did too*” contains an unpronounced syntactic structure (e.g., Fiengo & May, 1994; Merchant, 2001, 2015; Ross, 1969; Sag, 1976; Yoshida et al., 2015). This elided structure is resolved by material in the preceding context that is syntactically and/or semantically identical to it (e.g., Chomsky, 1965; Chung et al., 1995; Fiengo & May, 1994; Hankamer & Sag, 1976; Merchant, 2001, 2008, 2013; Yoshida, 2010). According to this analysis, in (1), “*did too*” contains an inaudible verb phrase structure, which is resolved by the antecedent “*liked Mary*” that is syntactically (vP) and semantically (λx.liked(x, Mary)) identical to it (Fig. 1).

Ellipsis has been widely investigated in sentence processing studies. These studies have addressed questions such as (i) whether identity between the elided material and its antecedent affects sentence comprehension (e.g., Arregui et al., 2006; Dickey & Bunger, 2011; Tanenhaus & Carlson, 1990), (ii) whether antecedent complexity influences ellipsis resolution (e.g., Frazier & Clifton, 2000; H. Kim et al., 2025; Martin & McElree, 2008; Murphy, 1985), (iii) how the processor accesses information about an antecedent to resolve the elided material (e.g., N. Kim et al., 2019; Snider & Runner, 2010), and (iv) how ellipsis ambiguities are resolved (e.g., Carlson, 2001, 2002; Frazier & Clifton, 1998, 2005; N. Kim et al., 2020; Yoshida, Lee, et al., 2013). One issue that remains unresolved concerns the real-time resolution of VP ellipsis ambiguities:

- (2) Bill liked himself, and John did too.
- (3a) Strict interpretation
- Bill liked himself, and John *liked Bill*.
- (3b) Sloppy interpretation
- Bill liked himself, and John *liked himself*.

Sentence (2) contains a reflexive (“*himself*”), which corefers with “*Bill.*” With the reflexive in the antecedent, “*did too*” allows for either the strict interpretation (3a) or the sloppy interpretation (3b) (e.g., Büring, 2005; Keenan, 1971; Reinhart, 1983; Sag, 1976; Williams, 1977).

Numerous studies have investigated whether the strict or sloppy interpretation is preferred using offline measures, and these studies have yielded largely consistent results. Specifically, a preference for the sloppy interpretation has been widely observed in sentences like (2) (e.g., Frazier & Clifton, 2000; Gandón-Chapela & Gallardo-del-Puerto, 2025; Gezen, 2022; C. Kim & Runner, 2011; Ong & Brasoveanu, 2014; Storoshenko & Weir, 2022), although this preference is not often absolute or a preference for the strict interpretation has also been reported (e.g., Hall, 2021).

In contrast, relatively few studies have examined the real-time resolution of VP ellipsis ambiguities, and their findings have been inconclusive. These studies suggest that the strict interpretation is computed during ellipsis ambiguity resolution (Shapiro & Hestvik, 1995; Shapiro et al., 2003), or that there is only a weak preference for either the strict (Hall, 2021) or sloppy (Frazier & Clifton, 2000) interpretation. The present study aims to clarify the processor’s preference in the real-time resolution of VP ellipsis ambiguities and to identify potential causes of the inconclusive findings in the literature.

Our study also aims to examine whether a form mismatch between the elided material and its antecedent affects sentence processing. In VP ellipsis, the elided material does not need to be isomorphic to its antecedent. For example, in (4), the sloppy interpretation—“*Mary liked herself*”—is possible even though the antecedent does not contain “*herself.*”

- (4) Bill liked himself, and Mary did too.

When the processor encounters “*did too*” in (4), it needs to retrieve information about “*liked himself*” from memory to resolve the elided material. This resolution may incur increased processing costs due to a gender mismatch between “*Mary*” and “*himself*” (e.g., Sturt, 2003), or due to the shift from “*himself*” to “*herself.*” Although a few studies have examined whether such mismatches affect sentence comprehension (Aparicio et al., 2015; Gandón-Chapela & Gallardo-del-Puerto, 2025; Gezen, 2022), aside from Hall (2021), none have investigated their influence on real-time ambiguity resolution in sentences like (4).

In this paper, we report three reading experiments (Experiments 1–3) designed to examine whether the previous inconclusive findings could be attributed to: (i) insufficient statistical power, (ii) limitations in experimental materials, or (iii) the influence of processing depth. In what follows, we first review previous research on VP ellipsis and the resolution of VP ellipsis ambiguities.

## VP ellipsis and ambiguity resolution

The elided material in (2) can be interpreted in two different ways (3a/b). To allow for these interpretations, the conditions on identity between the elided material and its antecedent need to be relaxed. In the sloppy interpretation, the elided material and its antecedent are isomorphic (i.e., “*liked himself*”), but referential information (indicated in (5a/b) as 1 or 2) must differ. The strict interpretation would require that “*himself*” is reconstructed as a pronominal (“*him*”) at the ellipsis site (Fiengo & May, 1994).^1^

- (5a) Strict interpretation
- [_NP1_ Bill] liked [_NP1_ himself], and [_NP2_ John] liked [_NP1_ him].
- (5b) Sloppy interpretation
- [_NP1_ Bill] liked [_NP1_ himself], and [_NP2_ John] liked [_NP2_ himself].

While the strength of the processor’s preference for, or the availability of, the strict and sloppy interpretations may be influenced by various factors (e.g., Frazier & Clifton, 2000; Hestvik, 1995; Kehler, 2000; C. Kim & Runner, 2011; Ong & Brasoveanu, 2014), previous offline studies have generally observed a preference for the sloppy interpretation in sentences like (2). However, this preference is often not absolute. For example, C. Kim and Runner (2011) presented participants with sentences like (2), and asked questions such as “*Who did John like?*” with two answer options: “*Bill*” and “*John.*” Selecting “*Bill*” indicates a preference for the strict interpretation, while selecting “*John*” indicates a preference for the sloppy interpretation. Their results showed that approximately 65% of responses favoured the sloppy interpretation, while about 35% favoured the strict interpretation.

In contrast, research on real-time processing has produced inconclusive findings. Shapiro and Hestvik (1995) investigated whether the strict interpretation is computed at an ellipsis site using a cross-modal lexical priming task. In this task, participants listened to sentences like (6).

- (6) The policeman defended himself, and the fireman did too,

At the moment participants heard the word “*did,*” a word was displayed on a computer screen. This word was either semantically related (*robber*) to “*The policeman*” or unrelated (*roller*). Participants needed to quickly judge whether the word was a real word. If the strict interpretation (“*the fireman defended the policeman*”) is computed, lexical decisions are expected to be faster for semantically related words than for semantically unrelated words due to the reactivation of “*The policeman.*” Shapiro and Hestvik observed this reactivation effect, suggesting that the strict interpretation is computed at “*did too.*”

Frazier and Clifton (2000) tested sentences like (7a/b) to investigate how VP ellipsis ambiguities are resolved.

- (7a) John [_antecedent_ thinks it’s a good idea to shave himself before he goes to sleep] and Andy [_ellipsis_ does too].
- (7b) John [_antecedent_ thinks it’s a good idea to shave himself before he goes to sleep] and Anne [_ellipsis_ does too].

In (7a-b), “*shave himself*” in the antecedent would be a more natural action for the male name “*Andy*” (7a) than for the female name “*Anne*” (7b). Thus, if there is a preference for the sloppy interpretation, longer reading times at “*does too*” are expected in (7b). Frazier and Clifton observed this reading time pattern, although only numerically (1,044 ms vs. 1,097 ms; no statistical analyses are reported). This finding potentially suggests a preference for the sloppy interpretation.

Hall (2021) investigated the processor’s preference in resolving VP ellipsis ambiguities using sentences like (8a/b):

- (8a) [_context_ Although John didn’t punish himself], Bill [_antecedent_ punished himself], and John [_ellipsis_ did too] since it was necessary.
- (8b) [_context_ Although John didn’t punish him], Bill [_antecedent_ punished himself], and John [_ellipsis_ did too] since it was necessary.

In (8a/b), the first clause provides context indicating that John did not punish himself or Bill (him). When the first clause contains “*himself*” (8a), the sloppy interpretation at “*did too*” (“*John punished himself*”) becomes incompatible with the context. Conversely, When the first clause contains “*him*” (8b), the strict interpretation (“*John punished Bill*”) becomes incompatible. Such incompatibilities are expected to cause processing difficulty (e.g., Cunnings & Fujita, 2021; Frazier & Rayner, 1982; Fujita & Cunnings, 2020, 2021a; Sturt et al., 1999). Therefore, if the processor prefers either interpretation, processing times at “*did too*” should vary accordingly.

Hall also manipulated the gender of the subjects in the context and ellipsis clauses to examine the influence of form mismatches:

- (8c) [_context_ Although Mary didn’t punish herself], Bill [_antecedent_ punished himself], and Mary [_ellipsis_ did too] since it was necessary.
- (8d) [_context_ Although Mary didn’t punish him], Bill [_antecedent_ punished himself], and Mary [_ellipsis_ did too] since it was necessary.

The idea is that, when information about “*punished himself*” is retrieved from memory at “*did too,*” processing costs may increase in (8c/d) due to a gender mismatch between “*himself*” and “*Mary*” (e.g., Fujita, 2021, 2024b; Fujita & Cunnings, 2021b; Hall & Yoshida, 2021; Kazanina et al., 2007; Kush et al., 2017; Schneider & Phillips, 2001; Sturt, 2003), or due to the cost of shifting from “*himself*” to “*herself.*”^2^

In an eye-tracking experiment, Hall observed only weak evidence of longer reading times in the “*him*” conditions than in the “*himself*” condition (*p* =.07, only in total viewing times at the spillover region), suggesting a weak preference for the strict interpretation. Hall also found a gender mismatch effect when the first clause enforced the sloppy interpretation (i.e., in the “*him*” condition); however, this effect was only present at the second spillover region, “*it was.*”

In summary, studies on real-time sentence processing have produced inconclusive findings. Frazier and Clifton (2000) reported a preference for the sloppy interpretation, whereas Hall (2021) found evidence suggesting a preference for the strict interpretation. Crucially, the evidence presented in both studies is weak. Additional support for the computation of the strict interpretation has been reported by Shapiro and Hestvik (1995). These findings suggest that there may be no consistent preference for either the strict or sloppy interpretation, or that certain factors may influence the processor’s preference during real-time processing. Furthermore, little is known about the influence of form mismatches on real-time ellipsis ambiguity resolution.

## General methods

### The present study

We report three reading experiments (Experiments 1–3) designed to investigate potential causes of the inconclusive findings about the real-time resolution of VP ellipsis ambiguities in previous research. These experiments also examined the influence of form mismatches. For Experiments 1–3, we adopted the research design (8a–d) used in Hall (2021) because it allowed us to directly test the processor’s preference in VP ellipsis ambiguity resolution and the influence of form mismatches.

Experiment 1 used the same experimental sentences as Hall (2021), but with a substantially larger sample size. The aim of this replication attempt was to determine whether the weak evidence reported by Hall was due to insufficient statistical power. Given the inconclusive findings in previous research, any preference in VP ellipsis ambiguity resolution might be subtle—that is, the effect of interpretation in (8a–d) could be very small. Hall recruited 52 participants, which may be insufficient to detect such a small effect given previous research on real-time sentence processing (e.g., Fujita, 2024a, 2025a; Jäger et al., 2020; Parker, 2019; Vasishth et al., 2018). To address this, Experiment 1 tested a large sample size (360 participants) to obtain more reliable results.

Experiment 2 used sentences like those in Experiment 1, but with some modifications. Specifically, Experiment 2 used the present tense to enforce a habitual reading for an action verb, as exemplified below.

- (9) John never punishes himself/Bill, but Bill punishes himself, and John does too all the time.

The original sentences used the past tense (e.g., “*John didn’t punish…*”), which could allow the events described in the context and ellipsis clauses to occur in different times. For example, in (8a), the event in the context clause might be interpreted as occurring at a different time than the event in the ellipsis clause, as in *Although John didn’t punish himself/Bill at one time, John punished himself/Bill at another*. Under this interpretation, the context clause is not inconsistent with either the strict or sloppy interpretation. Using the present tense enforces a habitual reading in (9), which makes the context clause clearly inconsistent with either the strict (“*John punishes Bill*”) or sloppy (“*John punishes himself*”) interpretation. Experiment 2 addressed this methodological concern.

Experiment 3 investigated whether the inconclusive findings could be attributed to the influence of processing depth. Previous research suggests that processing depth affects ambiguity resolution (e.g., Dwivedi, 2013; Ferreira & Patson, 2007; Sanford & Sturt, 2002; Stewart et al., 2007; Sturt et al., 2004; Swets et al., 2008). To examine this influence, Experiment 3 included comprehension questions targeting the interpretation of the elided material (e.g., “*What does John do all the time?*” 1. “*Punishes himself,*” 2. “*Punishes Bill*” for (9)). The experiment explored whether preferences for the strict or sloppy interpretation varied depending on comprehension accuracy.

## Experiment 1

### Methods

Experiment 1 is a replication study. Using the same experimental sentences as Hall (2021) but with a larger sample size, this experiment investigated whether a clear preference for either the sloppy or strict interpretation would emerge and whether form mismatches would affect real-time ambiguity resolution. A sample set of experimental sentences is provided below.

- (10a) Strict interpretation, gender match
- Although John didn’t punish himself, Bill punished himself, and John did too since it was necessary.
- (10b) Strict interpretation, gender mismatch
- Although Mary didn’t punish herself, Bill punished himself, and Mary did too since it was necessary.
- (10c) Sloppy interpretation, gender match
- Although John didn’t punish him, Bill punished himself, and John did too since it was necessary.
- (10d) Sloppy interpretation, gender mismatch
- Although Mary didn’t punish him, Bill punished himself, and Mary did too since it was necessary.

In (10a–d), the third clause contains an ellipsis site (“*did too*”), and “*punished himself*” in the second clause serves as the antecedent for interpreting the elided material. The ellipsis site allows for either the strict (“*John/Mary punished Bill*”) or sloppy (“*John/Mary punished himself/herself*”) interpretation. However, in (10a/b), the first clause provides a context that contradicts the sloppy interpretation (“*John/Mary didn’t punish himself/herself*”). Conversely, in (10c/d), the first clause contradicts the strict interpretation (“*John/Mary didn’t punish him (= Bill)*”). Therefore, if there is a preference for the strict interpretation, longer reading times are expected in (10c/d) than in (10a/b). In contrast, a preference for the sloppy interpretation should result in longer reading times in (10a/b).

The sentences in (10a–d) also test the influence of form mismatches. In (10a/c), the subject of the ellipsis clause matches the gender of the reflexive (“*John,*” “*himself*”), whereas in (10b/d), there is a mismatch (“*Mary,*” “*himself*”). In (10b/d), this manipulation may cause processing difficulty at “*did too*” due to a gender mismatch between “*Mary*” and “*himself*” (Fujita & Yoshida, 2024; Sturt, 2003), or due to the cost of shifting from “*himself*” to “*herself.*”

#### Participants

For Experiment 1, we collected data from 360 native English speakers (199 women, 161 men; mean age = 36 years, *SD* = 10, range: 20–60) recruited via Prolific (https://www.prolific.com/). All participants were British citizens who had lived in the UK for most of their lives before the age of 18.

#### Materials

The materials consisted of 24 sets of experimental sentences like (10a–d), 72 filler sentences, and practice sentences. None of the filler sentences contained VP ellipsis.

#### Procedures

Reading times were measured using the L-maze task (Boyce et al., 2020; Fujita, 2025c; Witzel et al., 2012). The task was implemented using Ibex (https://github.com/addrummond/ibex) and administered in PCIbex Farm (Zehr & Schwarz, 2018). In this task, participants read each sentence word by word, with each word presented alongside a pseudoword. Participants were instructed to press the button corresponding to the word that continued the sentence. If a pseudoword was selected, the trial ended, and a warning message appeared before the next trial began. Experimental sentences were presented in a Latin square design and in a pseudo-randomised order. The task began with several practice trials.

#### Data analysis

The outcome variable was log-transformed reading times at the ellipsis (“*did too*”) and spillover (“*since*”) regions. These reading times were analysed by fitting linear mixed-effects models using *lme4* (Bates et al., 2015) in R (R Core Team, 2025). The reading time at the ellipsis region was defined as the sum of the reading times at “*did*” and “*too.*” Predictor variables were interpretation (strict/sloppy), gender (match/mismatch), and their interaction. These predictors were sum-coded (−1/+1). Each model was initially fitted with participant- and item-specific varying intercepts and varying slopes for all predictor variables, as well as correlation parameters among these participant/item-specific coefficients. When the initial model failed to converge, we first set the correlation parameters to zero. If the model still did not converge, we simplified the model by iteratively removing the participant- or item-specific slope term with the smallest estimated variance component, refitting after each step, until convergence was achieved.

### Results

**Fig. 2:**
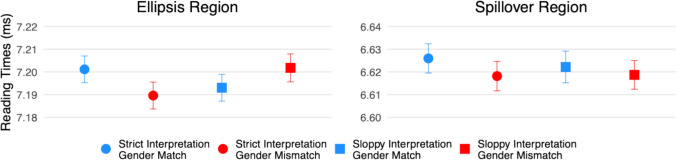
Log-transformed reading times at the ellipsis (“did too”) and spillover (“since”) regions in Experiment 1. (Colour figure online)

**Fig. 3:**
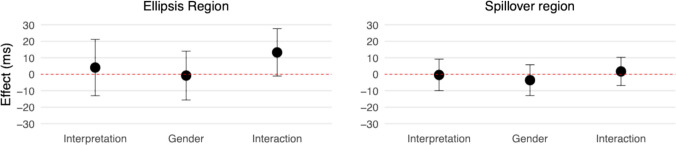
Effect estimates and 95% compatibility intervals in Experiment 1

Figure 2 presents log-transformed reading times, while Fig. 3 displays effect estimates with 95% compatibility intervals (CIs). Although reading times were analysed in log ms, the effect estimates reported below are back transformed.

#### Ellipsis region (“did too”)

There were no clear effects of interpretation (95% CI [−13, 21] ms, *t* = 0.47), gender (95% CI [−16, 14] ms, *t* = −0.11), or their interaction (95% CI [−1, 28] ms, *t* = 1.81).

#### Spillover region (“since”)

Similarly, no clear effects were observed for interpretation (95% CI [−10, 9] ms, *t* = −0.08), gender (95% CI [−13, 6] ms, *t* = −0.75), or their interaction (95% CI [−7, 10] ms, *t* = 0.40).

### Summary of Experiment 1

Experiment 1 did not reveal any clear effects. This result is not incompatible with Hall (2021), who tested a small sample and found only very weak evidence for a strict-interpretation preference and for form mismatch effects. Regarding real-time ambiguity resolution, the results of Experiment 1 may suggest that the processor does not have a strong preference for either the strict or sloppy interpretation.

However, as discussed earlier in this paper, the absence of interpretation effects might be attributed to the use of the past tense. The past tense may allow the processor to infer that the events described in the context clause and the ellipsis clause occurred at different times, thereby reducing the perceived inconsistency between the two clauses. To address this concern, Experiment 2 used the present tense instead of the past tense. The present tense enforces habitual interpretations of the action verbs in the experimental materials, which should make the inconsistency between the context clause and either the strict or sloppy interpretation more apparent.

## Experiment 2

### Methods

Experiment 2 tested sentences like those used in Experiment 1, but in the present tense. A sample set of experimental sentences is provided below.

- (11a) Strict interpretation, gender match
- John never punishes himself, but Bill punishes himself, and John does too all the time.
- (11b) Strict interpretation, gender mismatch
- Mary never punishes herself, but Bill punishes himself, and Mary does too all the time.
- (11c) Sloppy interpretation, gender match
- John never punishes Bill, but Bill punishes himself, and John does too all the time.
- (11d) Sloppy interpretation, gender mismatch
- Mary never punishes Bill, but Bill punishes himself, and Mary does too all the time.

The use of the present tense enforces a habitual reading in (11a–d), making it clear that the first clause (i.e., “*John/Mary never punishes himself/Bill*”) contradicts either the strict (i.e., “*John/Mary punishes Bill*”) or sloppy (“*John/Mary punishes himself/herself*”) interpretation. Additionally, Experiment 2 used proper names (“*Bill*”) in the first clauses of the sloppy-interpretation conditions (11c/d), instead of pronominals (“*him*”) used in (10c/d) in Experiment 1 (“*Although John/Mary didn’t punish him, Bill…*”), to avoid referential ambiguity.

The predictions for Experiment 2 were like those for Experiment 1. If there is a preference for the strict interpretation, reading times at the ellipsis site should be longer in (11c/d) than in (11a/b). Conversely, a preference for the sloppy interpretation should result in longer reading times in (11a/b). Regarding the influence of form mismatches, no effect is expected given the results of Experiment 1.

#### Participants

For Experiment 2, we collected data from 160 native speakers of British English (90 women, 69 men; mean age = 37 years, *SD* = 10, range: 20–60; information from one participant could not be obtained) recruited via Prolific. None of these participants had taken part in Experiment 1.

#### Materials

The materials for Experiment 2 were 24 sets of experimental sentences like (11a–d), 72 filler sentences, and practice sentences.

#### Procedures and data analysis

The procedure and data analysis were identical to those in Experiment 1.

### Results

**Fig. 4:**
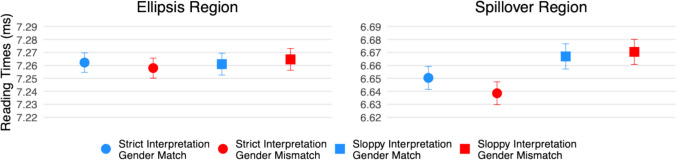
Log-transformed reading times at the ellipsis (“does too”) and spillover (“all”) regions in Experiment 2. (Colour figure online)

**Fig. 5:**
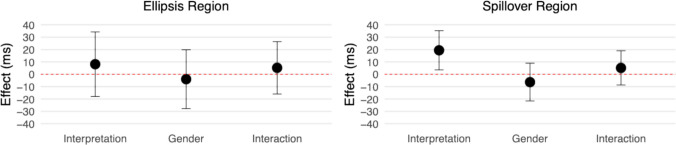
Effect estimates and 95% compatibility intervals in Experiment 2

Log-transformed reading times are shown in Fig. 4, and effect estimates are presented in Fig. 5.

#### Ellipsis region (“does too”)

There were no clear effects of interpretation (95% CI [−8, 41] ms, *t* = 0.61), gender (95% CI [−22, 26] ms, *t* = −0.33), or their interaction (95% CI [−11, 27] ms, *t* = 0.48).

#### Spillover region (“all”)

There was a main effect of interpretation (95% CI [4, 35] ms, *t* = 2.40), with longer reading times in the sloppy-interpretation conditions than in the strict-interpretation conditions. No clear effects were found for gender (95% CI [−22, 9] ms, *t* = –0.82) or the interaction (95% CI [−9, 19] ms, *t* = 0.73).

### Summary of Experiment 2

Experiment 2 yielded two key findings. First, there was no evidence that gender mismatch influenced sentence processing, replicating the results of Experiment 1 regarding form mismatches. Second, reading times at the spillover region were longer in the sloppy-interpretation conditions than in the strict-interpretation conditions. This reading time pattern indicates a preference for the strict interpretation, suggesting that the lack of a clear preference observed in Experiment 1 may be due to the use of the past tense.

Experiment 2 revealed a preference for the strict interpretation during real-time sentence processing. We now turn to the final experiment—Experiment 3—which aimed to explore whether processing depth is a cause of the contradictory or inconclusive results reported in previous research. Recall that Frazier and Clifton (2000) reported very weak evidence supporting a preference for the sloppy interpretation, whereas Hall (2021) observed a very weak preference for the strict interpretation. Furthermore, although Experiment 2 showed a relatively clear preference for the strict interpretation, this preference was only observed at the spillover region and was small in magnitude (approximately 20 ms). While delayed effects are not uncommon in sentence processing research, the L-maze task used in Experiment 2 typically reveals well-localised effects (e.g., Boyce et al., 2020; Fujita, 2025c; Fujita & Yoshida, 2024; Han et al., 2025; Witzel et al., 2012).

Previous research has argued or observed that processing depth influences ambiguity resolution (e.g., Dwivedi, 2013; Ferreira & Patson, 2007; Sanford & Sturt, 2002; Stewart et al., 2007; Sturt et al., 2004; Swets et al., 2008). For example, Swets et al. (2008) examined globally ambiguous sentences involving relative clauses, such as “*The maid of the princess who scratched herself…*”, where the relative clause “*who scratched herself*” can modify either the first nominal (“*The maid…*”) or the second nominal (“*the princess*”). These sentences were compared with two types of locally ambiguous sentences: one where the relative clause modified the first nominal due to gender agreement (e.g., “*The maid of the prince who scratched herself…*”) and another where it modified the second nominal (“*The son of the princess who scratched herself…*”). Some studies have shown that globally ambiguous sentences are read more quickly than locally ambiguous sentences (e.g., Traxler et al., 1998; van Gompel et al., 2001). However, Swets et al. observed that this ambiguity advantage disappears when participants are asked about the interpretation of the relative clause (e.g., “*Did the maid/princess/son scratch?*”). This finding suggests that real-time ambiguity resolution processes can vary depending on the degree to which readers attend to ambiguities.

To investigate the influence of processing depth on VP ellipsis ambiguity resolution, Experiment 3 included comprehension questions designed to assess whether participants correctly resolved VP ellipsis ambiguities. Comprehension accuracy was then included as a predictor in the statistical models to test whether it predicted processing times at the ellipsis site. There are several possible ways in which processing depth may influence ambiguity resolution. For example, our experimental manipulation (e.g., “*John never punishes himself/Bill, but Bill punishes himself, and John does too…*”) requires participants to notice an inconsistency between the first clause (“*John never punishes himself/Bill*”) and the ellipsis clause (“*John does too*”). It is possible that participants become aware of this inconsistency only when they attend to the ellipsis site. In this case, we would expect clear interpretation effects only when comprehension questions are answered correctly.

## Experiment 3

### Methods

Experiment 3 used the same materials as in Experiment 2, with the addition of comprehension questions probing the interpretation of the elided material.

- (12a) Strict interpretation, gender match
- John never punishes himself, but Bill punishes himself, and John does too all the time.
- (12b) Strict interpretation, gender mismatch
- Mary never punishes herself, but Bill punishes himself, and Mary does too all the time.
- (12c) Sloppy interpretation, gender match
- John never punishes Bill, but Bill punishes himself, and John does too all the time.
- (12d) Sloppy interpretation, gender mismatch
- Mary never punishes Bill, but Bill punishes himself, and Mary does too all the time.

Comprehension question:

What does John/Mary do all the time?

1. Punishes Bill
2. Punishes himself/herself

Each comprehension question was presented with two answer options: one consistent with the strict interpretation (“*John/Mary punishes Bill*”) and one consistent with the sloppy interpretation (“*John/Mary punishes himself/herself*”). As noted, one possible way in which processing depth influences real-time ambiguity resolution is that participants become aware of an inconsistency between the first clause and the ellipsis clause only when they attend to the ellipsis site. In this case, a preference for the strict or sloppy interpretation may emerge only when participants answer comprehension questions correctly.

#### Participants

For Experiment 3, we collected data from 308 native speakers of British English (187 women, 120 men, one preferred not to say; mean age = 37 years, *SD* = 10, range: 19–60) via Prolific. None of these participants had taken part in Experiments 1 or 2.

#### Materials

The materials consisted of 24 sets of experimental sentences like (12a–d), 72 filler sentences, and practice sentences. Each sentence was followed by a comprehension question with two answer options. For the experimental sentences, questions always probed the interpretation of the elided material. The order of the answer options was randomised to balance the position of the correct answer across the strict- and sloppy-interpretation conditions.

#### Procedures

The procedure was the same as in Experiments 1 and 2, with one key difference: Experiment 3 used a noncumulative, word-by-word self-paced reading task. In this task, at the start of each trial, participants saw dashes masking the entire sentence. Pressing the space bar revealed the first word, and each subsequent key press revealed the next word while masking the previous word. After reading the final word and pressing the space bar, participants were presented with a comprehension question, which they answered by pressing either the “1” (first option) or “2” (second option) key.

We used self-paced reading rather than L-maze because combining L-maze with comprehension questions may impose excessive task demands on participants, although some studies suggest that this combination is feasible (e.g., Han et al., 2025). In addition, comprehension questions are more commonly used with self-paced reading, and we considered it reasonable to follow this standard practice.

#### Data analysis

Experiment 3 had two outcome variables: reading times and comprehension accuracy, which were analysed separately. The analysis of reading time data followed the same procedure as in Experiments 1 and 2. However, the linear mixed-effects models in Experiment 3 included an additional predictor—accuracy—indicating whether participants answered comprehension questions for experimental sentences correctly. This predictor was sum-coded.

Comprehension accuracy was analysed using logistic regression with generalised linear mixed-effects models. These models included interpretation (strict/sloppy), gender (match/mismatch), and their interaction as predictors.

### Results

Mean accuracy for filler questions was 91% (range: 72–100%), indicating that participants generally paid attention to the task.

#### Comprehension accuracy

**Fig. 6:**
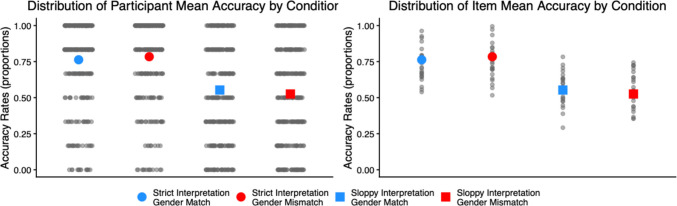
Comprehension accuracy in Experiment 3. Blue and red dots indicate the mean accuracy for each experimental condition. Small grey dots represent individual means, either by participant (left) or by item (right). (Colour figure online)

Figure 6 illustrates the mean accuracy rates for experimental questions in each condition, along with the distributions of participant-level and item-level mean accuracy.

The model revealed a main effect of interpretation (95% CI [−36, −24] %, *z* = −9.03), with higher accuracy in the strict-interpretation conditions than in the sloppy-interpretation conditions. Although there was a two-way interaction (95% CI [−6, −1] %, *z* = −2.92), a follow-up analysis showed similar effects of interpretation in the gender-match (95% CI [−33, −20] %, *z* = 7.60) and gender-mismatch (95% CI [−39, −27] %, *z* = 10.06) conditions.

This interaction was driven by differences in the effect of gender. For the strict-interpretation conditions, accuracy was higher in gender-mismatch sentences than in gender-match sentences (95% CI [0.05, 7] %, *z* = 1.99). By contrast, for the sloppy-interpretation conditions, accuracy was higher in gender-match sentences than in gender-mismatch sentences (95% CI [−6, −0.3] %, *z* = −2.17).

#### Reading times

**Fig. 7:**
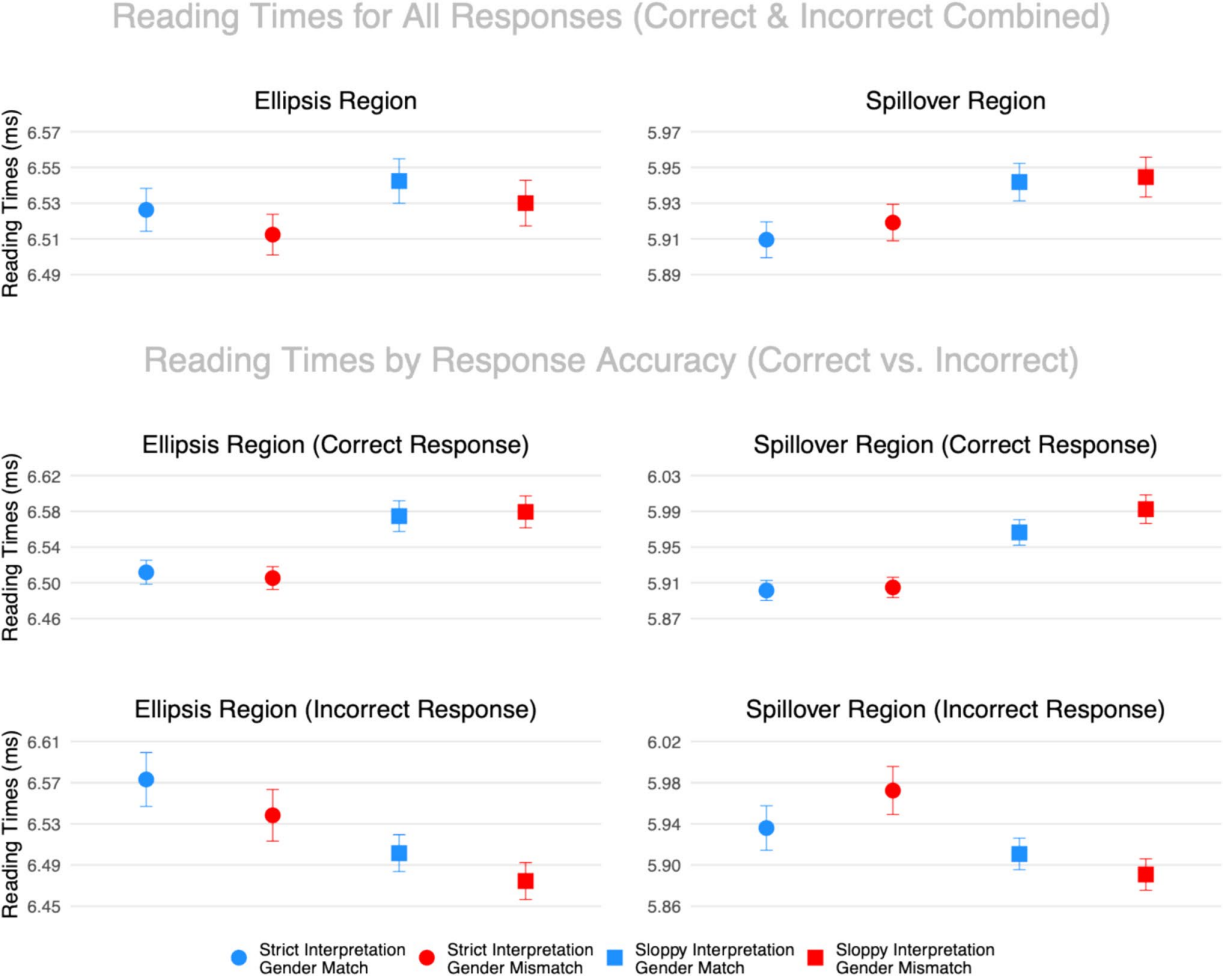
Log-transformed reading times at the ellipsis (“does too”) and spillover (“all”) regions in Experiment 3. The top two graphs show reading times for all responses, whereas the bottom four graphs show reading times for correct and incorrect responses separately. (Colour figure online)

**Fig. 8:**
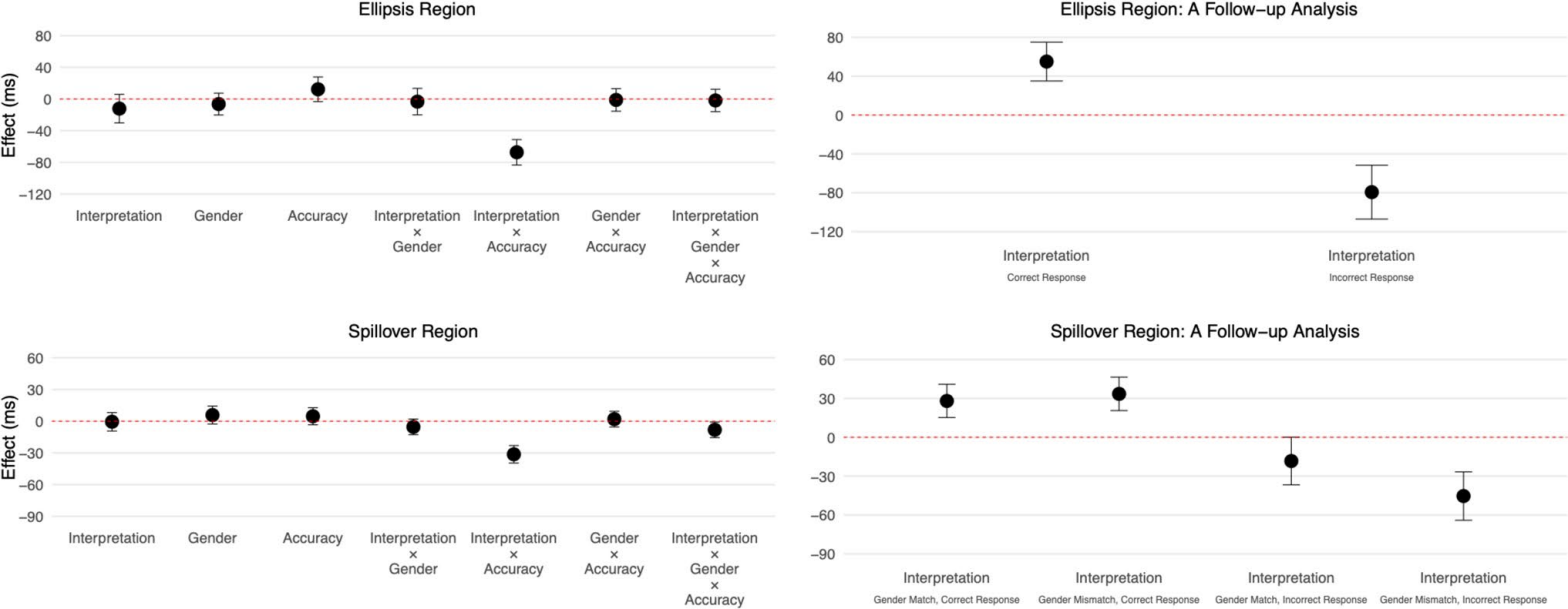
Effect estimates and 95% compatibility intervals in Experiment 3

Log-transformed reading times are shown in Fig. 7, and effect estimates are presented in Fig. 8.

#### Ellipsis region (“does too”)

There was an interaction between interpretation and accuracy (95% CI [−83, −51] ms, *t* = −8.22). This interaction was examined by analysing the effect of interpretation within each level of accuracy (correct or incorrect response). This analysis revealed contrasting patterns. Specifically, when questions were answered correctly, reading times were longer in the sloppy-interpretation conditions than in the strict-interpretation conditions (95% CI [35, 75] ms, *t* = 5.40), indicating a preference for the strict interpretation. Conversely, when questions were answered incorrectly, reading times were longer in the strict-interpretation conditions (95% CI [−107, −52] ms, *t* = −5.62), indicating a preference for the sloppy interpretation. The models did not show any other effects (see Fig. 8).

#### Spillover region (“all”)

The results were like those of the ellipsis region. There was an interaction between interpretation and accuracy (95% CI [−40, −23] ms, *t* = −7.40). A follow-up analysis indicated longer reading times in the sloppy-interpretation conditions when questions were answered correctly (95% CI [21, 41] ms, *t* = 6.17), and longer reading times in the strict-interpretation conditions when questions were answered incorrectly (95% CI [−46, −18] ms, *t* = −4.48). Although the model also showed a three-way interaction between interpretation, gender, and accuracy (95% CI [−16, −1] ms, *t* = −2.16), the overall pattern—that processing preferences depend on comprehension accuracy—remained consistent (see Fig. 8).

### Summary of Experiment 3

Comprehension accuracy:

(i) Overall comprehension accuracy for experimental sentences was low (66%).
(ii) Mean comprehension accuracy was higher in the strict-interpretation conditions (77%) than in the sloppy-interpretation conditions (54%).
(iii) There was a small effect of gender: in the strict-interpretation conditions, comprehension accuracy was higher when there was a gender mismatch (76% vs. 78%), whereas in the sloppy-interpretation conditions, comprehension accuracy was higher when there was a gender match (55% vs. 53%).

Reading times:

(iv) When comprehension questions were answered correctly, there was a preference for the strict interpretation.
(v) When comprehension questions were answered incorrectly, there was a preference for the sloppy interpretation.

In the following section, we discuss the implications of the findings from Experiments 1–3.

## General discussion

This study had two primary aims: first, to investigate whether there is a preference for either the strict or sloppy interpretation during real-time sentence processing; and second, to explore whether form mismatches influence VP ellipsis ambiguity resolution. Below, we first discuss the implications of our findings for real-time sentence processing and then for offline sentence comprehension.

### Real-time sentence processing

Previous research has produced contradictory or inconclusive results regarding the real-time resolution of VP ellipsis ambiguities. We considered three potential causes for these results: (i) insufficient statistical power, (ii) limitations in the experimental materials, and (iii) the influence of processing depth.

Experiment 1 addressed the issue of statistical power. With a large sample size of 360 participants, this experiment revealed no clear preference, suggesting that insufficient power is unlikely to explain previous inconclusive findings.

Experiment 2, which used revised experimental sentences, revealed a relatively clear preference for the strict interpretation. This suggests that the weak evidence in Hall’s (2021) study may be due to shortcomings in its materials.

Experiment 3 revealed that processing depth influences how VP ellipsis ambiguities are resolved during real-time processing. When participants were more focused on sentence comprehension (i.e., when questions were answered correctly), there was a preference for the strict interpretation. Conversely, when they were less focused (i.e., when questions were answered incorrectly), a preference for the sloppy interpretation emerged. This finding can explain the previous results showing only a weak preference for either interpretation (e.g., Frazier & Clifton, 2000; Hall, 2021), and aligns with the finding that the strict interpretation is computed at both online and offline levels (e.g., C. Kim & Runner, 2011; Shapiro & Hestvik, 1995). The relatively clear, though not decisive, preference for the strict interpretation observed in Experiment 2 may be attributed to the use of the L-maze task, which requires focused attention for lexical judgements.

The present study also investigated the influence of form mismatches. Across three experiments, we observed no clear effect.

These findings raise the question of what processing mechanisms underlie real-time ambiguity resolution. We propose the following account. In the VP ellipsis sentence “*Bill punishes himself, and John does too,*” when information about “*punishes himself*” is retrieved at “*does too*” from memory to interpret the elided material, the reflexive is not retrieved as its surface form “*himself*” but as an abstract nominal expression (see also Johnson, 2014; Kratzer, 1998; Storoshenko & Weir, 2022). The richness of the information that this retrieved nominal carries depends on processing depth: greater attention leads to more detailed information. Specifically, when relatively little attention is paid, the retrieved nominal carries only a reflexive feature [+ref(lexive), −pro(nominal)] (e.g., Chomsky, 1982):

- (13) Bill punishes himself, and John does $[\text{punishes }{\mathrm{NP}}_{[+\mathrm{ref}, -\mathrm{pro}]}]$ too.

Since the retrieved nominal “*NP*_*[+ref, –pro]*_” is a reflexive, its interpretation depends on the subject of the second clause (i.e., the sloppy interpretation). If the subject is a masculine NP as in (13), “*NP*_*[+ref, –pro]*_” is assigned the feature [+mas(culine)] and corefers with it (14a). Conversely, if the subject is a feminine NP, “*NP*_*[+ref, –pro]*_” is assigned [+fem(inine)] and corefers with it (14b):

- (14a) [_NP1_ Bill] punishes [_NP1_ himself], and [_NP2_ John] does $[\text{punishes }{\mathrm{NP}2}_{[+\mathrm{ref}, -\mathrm{pro}]}^{[+\mathrm{mas}]}]$ too.
- (14b) [_NP1_ Bill] punishes [_NP1_ himself], and [_NP2_ Mary] does $[\text{punishes }{\mathrm{NP}2}_{[+\mathrm{ref}, -\mathrm{pro}]}^{[+\mathrm{fem}]}]$ too.

Gender and reference information can be assigned via memory retrieval: after “*[punishes NP*_*[+ref, –pro]*_*]*” is retrieved, structural information is used to retrieve the reflexive’s antecedent (Dillon, 2014; Dillon et al., 2013; Fujita, 2024a, 2025b; Fujita & Yoshida, 2024; Kush, 2013).

When relatively more attention is paid, the retrieved nominal carries not only a reflexive feature but also gender and reference information:

- (15) [_NP1_ Bill] punishes [_NP1_ himself], and [_NP2_ John] does $[\text{punishes }{\mathrm{NP}1}_{[+\mathrm{ref}, -\mathrm{pro}]}^{[+\mathrm{mas}]}]$ too.

Here, the retrieved nominal contains conflicting information: its reference links to “*Bill*” but it carries a reflexive feature. This conflict however can be resolved by pronominalisation—changing [+ref, –pro] to [–ref, +pro] (Fiengo & May, 1994):

- (16) [_NP1_ Bill] punishes [_NP1_ himself], and [_NP2_ John] does $[\text{punishes }{\mathrm{NP}1}_{[-\mathrm{ref}, +\mathrm{pro}]}^{[+\mathrm{mas}]}]$ too.

This results in a strict interpretation. This ambiguity resolution process illustrates how preferences in VP ellipsis ambiguity resolution depend on processing depth. It also explains why we did not observe a clear form mismatch effect: for the sloppy interpretation, form mismatch effects do not arise because the retrieved nominal lacks a gender feature; for the strict interpretation, they do not arise because the retrieved nominal carries reference information linking it to the subject of the antecedent clause.

## Offline sentence comprehension

**Fig. 9:**
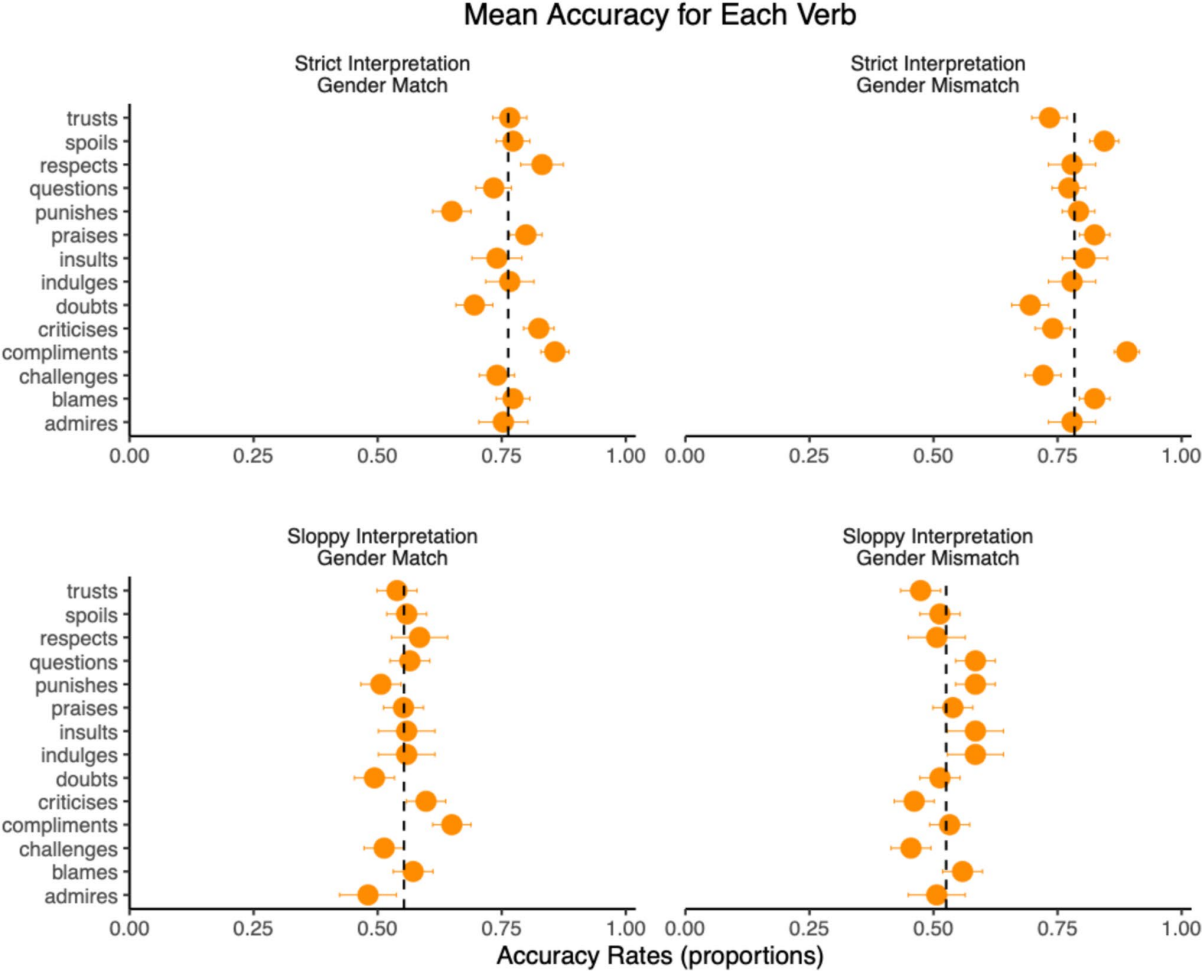
Mean accuracy rates for the verbs used in the antecedent clause in Experiment 3. The dotted vertical line marks the mean accuracy for each condition. Error bars are standard errors

Experiment 3 revealed that comprehension accuracy for experimental questions was low (mean: 66%), and this was mainly due to sloppy-interpretation sentences (strict interpretation: 77%; sloppy interpretation: 54%). The low accuracy in sloppy-interpretation sentences cannot be attributed to a lack of attention to the task, given that mean accuracy for filler questions was high (91%). Furthermore, as shown in Fig. 6, the distribution of item-level mean accuracy was relatively narrow, and similar distributions were observed across the verbs used in the antecedent (Fig. 9). Therefore, the low accuracy in sloppy-interpretation sentences does not appear to be driven by item-level effects.

Why was comprehension accuracy low in sloppy-interpretation sentences? One possibility is that it reflects differences in the difficulty of reinterpretation between strict- and sloppy-interpretation sentences. In strict-interpretation sentences (“*John never punishes himself, but Bill punishes himself, and John does too*”), if the processor initially computes the sloppy interpretation (“*John punishes himself*”), it must reinterpret the elided material as “*John punishes him (= Bill)*” for sentence comprehension. This reinterpretation involves pronominalisation discussed in the previous section.

In sloppy-interpretation sentences (“*John never punishes Bill, but Bill punishes himself, and John does too*”), if the processor initially computes the strict interpretation (“*John punishes him (= Bill)*”), it must make a reinterpretation to compute the sloppy interpretation (“*John punishes himself*”). This requires reflexivisation, which we propose is difficult, leading to low accuracy. Our proposal is supported by evidence that reflexivisation is not permissible in sentences like (17) (Safir, 2004a, 2004b).

- (17) Mary punishes [_NP1_ John], and [_*NP1/NP2_ he] does too.

The sentence in (17) becomes ungrammatical (*) when “*he*” corefers with “*John*”. However, if reflexivisation is possible, the sentence should be grammatical contrary to fact:

- (18) Mary punishes [_NP1_ John], and [_NP1_ he] punishes [_NP1_ himself] too.

As discussed, pronominalisation is possible in a similar sentence (e.g., “*Mary punishes Bill, and John does [*_*ellipsis*_* punishes him (= Bill)] too*”). Given this contrast, the lower accuracy in sloppy-interpretation sentences may reflect the difficulty of reinterpretation when reflexivisation is required.

Experiment 3 also showed slightly lower accuracy in strict-interpretation sentences when there was a gender match, and slightly lower comprehension accuracy in sloppy-interpretation sentences when there was a gender mismatch. Since Experiments 1–3 did not show a clear effect of gender on real-time processing, this small gender effect is likely attributable to post-interpretive processes.

One possible explanation for this finding concerns the influence of parallelism between the antecedent clause and the ellipsis clause. In the strict-interpretation sentence (19), the correct answer to (21) is “*Punishes Bill.*” However, when participants were presented with the incorrect option “*Punishes himself/herself,*” they may have been drawn to it in the gender-match condition due to surface-form parallelism (“*Bill punishes himself, and John punishes himself*”). Consequently, comprehension accuracy was lower when gender matched.

- (19) Strict interpretation
- John/Mary never punishes herself, but Bill punishes himself, and John/Mary does too…
- (20) Sloppy interpretation
- John/Mary never punishes Bill, but Bill punishes himself, and John/Mary does too…
- (21) What does John/Mary do all the time?
  1. Punishes Bill
  2. Punishes himself/herself

Conversely, in the sloppy-interpretation sentence (20), the correct answer is “*Punishes himself/herself.*” In this case, surface-form parallelism leads to higher accuracy, as observed in Experiment 3. Given substantial evidence that parallelism influences sentence processing (e.g., Carlson, 2002; Fujita, 2023; Yoshida, Dickey, et al., 2013a, 2013b), the gender effect observed in Experiment 3 may be attributed to surface-form parallelism between the antecedent and ellipsis clauses.

## Conclusion

Previous research has reported contradictory or inconclusive findings regarding how VP ellipsis ambiguities (e.g., “*Bill liked himself, and John did too*”) are resolved during real-time sentence processing, and there is limited evidence on whether form mismatches (e.g., “*Mary liked herself, and John did [*_*ellipsis*_* like himself] too*”) affect this process. To address these issues, we conducted three experiments.

Our results revealed no clear form mismatch effect and no evidence that previous inconclusive findings were due to insufficient statistical power. However, we observed that processing depth affects real-time ambiguity resolution: participants who correctly answered questions about the interpretation of the elided material tended to prefer the strict interpretation, whereas those who answered incorrectly favoured the sloppy interpretation.

To account for these findings, we proposed that reflexives are retrieved from memory as abstract nominals, and that greater attention to sentence processing leads to retrieval of more detailed information about the antecedent. These explain why form mismatches exert no robust influence and why preferences in VP ellipsis ambiguity resolution depend on processing depth.

## Data Availability

Data, analysis code, and experimental materials are available online (https://osf.io/t42b6).

## Footnotes

1 Some researchers argue that reflexives involve variable binding (e.g., Bill (λx (x liked x)); see Büring, 2005; Grodzinsky & Reinhart, 1993). In this paper, we discuss reflexive resolution from the perspective of coreference, because this view is familiar to readers of the literature most relevant to our study.

2 The acceptability of form mismatches can be explained in the same way as the strict interpretation—“*himself*” is reconstructed as “*herself*” at the ellipsis site.
